# David Charles Rayner

**Published:** 1945

**Authors:** 


### DAVID CHARLES RAYNER, Ch.M., F.R.C.S.

David Charles Rayner, a former president of the Medical Chirurgical Society, died in a Clifton Nursing Home on the 21st October, 1945.

He was a student of the Bristol Medical School, qualifying M.R.C.S., L.R.C.P., in 1892. He obtained the F.R.C.S. in 1896, became an original Fellow of the Royal College of Obstetricians and Gynæcologists in 1929, and received the Ch.M. degree from Bristol University in 1930. For a time he worked in the Physiological Department of the old University College in Bristol and from the outset of his professional career he specialized in Obstetrics and Gynæcology.

In 1897 he was elected to the staff of the Bristol General Hospital as Assistant Physician Accoucheur, an appointment he held for twenty-six years. It was during this period that he built up a large obstetrical and gynæcological practice in Bristol and district, where, by his industry, personal kindliness and charm, he established himself in the hearts of thousands of patients, and by his keenness and sound clinical teaching in the minds of countless medical students and pupil midwives. These nurses have special reason to remember his kindness and helpfulness, both as a teacher and as an examiner for the Central Midwives Board.

In the war of 1914–18 he worked on the staff of the Beaufort War Hospital at Fishponds, Bristol.

In 1929 he became Senior Obstetrician to the Bristol General Hospital under the title of Obstetric Physician and Gynæcologist. The nest step was in 1925, when the University of Bristol appointed him Director of Clinical Obstetrics, whilst a year later he became Professor of Obstetrics. During the session 1930–31, he was President of the Medical Chirurgical Society. In 1932 he was made Emeritus Professor of Obstetrics in recognition of his devoted service to the University and to his speciality.

He continued his private work, but in 1935, after a severe illness which necessitated the amputation of his right leg, his activities were naturally decreased. Even so, he remained willing to help whenever he could, and during the second World War he gave assistance by teaching and examining for the Central Midwives Board.

For many years he was a member of the Gynæcological Visiting Society, rarely missing a meeting, and paying visits to many teaching centres in Great Britain and on the Continent.

At the Annual Meeting of the British Medical Association held at Bath in 1925, he served as Vice-President of the Section of Obstetrics and Gynæcology, and continued his membership of the British Medical Association from 1894 until the time of his death, a period of continuous membership that can seldom have been exceeded.

To achieve a nickname has often been regarded as the hallmark of greatness and personality, and to former students and nurses of the Bristol General Hospital, Rayner was always known affectionately as "Charlie." Every one wondered how he sustained such a constant strain night and day over so many years, yet always appeared kindly and courteous, never ruffled or irritable. There is evidence to show that he had the "gift of sleep," and could refresh himself by odd hours snatched between appointments in his busy practice. In his lighter moments he derived great pleasure from his membership of the Bristol Medical Reading Society, to which he was elected as far back as the 3rd January, 1912. He seldom missed a meeting in those thirty-three years and was its secretary on two occasions, in 1913 and 1928.

His repution throughout Bristol and the West Country was founded upon hard work, willingly undertaken and conscientiously performed. His diffident, shy manner was no pose, his modest bearing represented the human kindliness of his generation. Generous to a fault in all his dealings, it is little wonder that his patients had implicit faith in his ability and experience. One whose name was for so long a household word in this city has passed and we, who were privileged to know and work under him, mourn the loss of a great gentleman whose long life of notable service will always be remembered.

